# Phase I studies of BI 1015550, a preferential phosphodiesterase 4B inhibitor, in healthy males and patients with idiopathic pulmonary fibrosis

**DOI:** 10.1183/23120541.00240-2022

**Published:** 2022-10-24

**Authors:** Toby M. Maher, Christina Schlecker, Doreen Luedtke, Sebastian Bossert, Donald F. Zoz, Armin Schultz

**Affiliations:** 1 Inflammation, Repair, and Development Section, National Heart and Lung Institute, Imperial College London, London, UK; 2 Keck Medicine of USC, Los Angeles, CA, USA; 3 Boehringer Ingelheim International GmbH, Ingelheim am Rhein, Germany; 4 Boehringer Ingelheim Pharma GmbH & Co. KG, Biberach an der Riss, Germany; 5 Boehringer Ingelheim Pharmaceuticals Inc, Ridgefield, CT, USA; 6 CRS Clinical Research Services Mannheim GmbH, Mannheim, Germany

## Abstract

**Introduction:**

BI 1015550 is a phosphodiesterase 4 (PDE4) inhibitor that has antifibrotic properties. Phase I and Ic studies were conducted to investigate the safety, tolerability and pharmacokinetics of BI 1015550 in healthy male subjects and patients with idiopathic pulmonary fibrosis (IPF).

**Methods:**

In the phase I study, 42 subjects were partially randomised to receive placebo or BI 1015550 in single rising doses of 36 mg and 48 mg, or multiple rising doses of 6 mg and 12 mg twice daily over 14 days. In the phase Ic study, 15 patients with IPF were randomised to receive 18 mg BI 1015550 or placebo twice daily for up to 12 weeks. For both studies, the primary endpoint was the number of subjects with drug-related adverse events (AEs).

**Results:**

In the Phase I study, drug-related AEs were reported for 50.0% of healthy male subjects treated with a single dose of BI 1015550, compared with 16.7% receiving placebo. For those receiving multiple doses, drug-related AEs were reported for 37.5% of those treated with BI 1015550 and 12.5% receiving placebo. The most frequently reported AEs by organ class were nervous system disorders, which were largely driven by headache. In the Phase Ic study, drug-related AEs were reported in 90.0% of patients treated with BI 1015550, compared with 60.0% of those receiving placebo. The most frequent AEs by organ class were gastrointestinal AEs.

**Conclusions:**

BI 1015550 had an acceptable safety profile in healthy male subjects and male and female patients with IPF, supporting further development in larger trials.

## Abstract 2

**A preferential PDE4B inhibitor, BI 1015550, is a candidate drug for the treatment of fibrotic interstitial lung disease. In Phase I studies, BI 1015550 had an acceptable safety profile in healthy males and patients with idiopathic pulmonary fibrosis.** https://bit.ly/3zSOtLs

## Introduction

Idiopathic pulmonary fibrosis (IPF) is a rare and fatal lung disease characterised by irreversible and progressive decline in lung function [1, 2]. There are two approved antifibrotic therapies for the treatment of IPF: nintedanib [3, 4] and pirfenidone [5, 6]. Nintedanib is approved for the treatment of IPF and other chronic fibrosing interstitial lung diseases (ILDs) with a progressive phenotype, and for systemic sclerosis-associated ILD [3, 4]. Pirfenidone is approved for the treatment of IPF [5, 6]. These treatments can slow, but not stop or reverse, disease progression and are associated with side effects that can delay treatment initiation or lead to discontinuation [7]. This means there is an unmet need for new treatments for IPF and other forms of progressive pulmonary fibrosis that can be used alone or with standard of care [8].

There are four phosphodiesterase 4 (PDE4) enzymes (PDE4A, B, C and D), which hydrolyse cyclic adenosine monophosphate to 5′adenosine monophosphate [9]. PDE4 is widely expressed in immune system cells, and inhibition of PDE4 reduces the release of pro-inflammatory mediators and the recruitment of inflammatory cells [10]. PDE4 inhibitors are associated with anti-inflammatory and antifibrotic effects, and have the potential to reduce pulmonary inflammation and fibrotic remodelling in lung diseases [8, 11]. However, the use of oral PDE4 inhibitors is limited due to their systemic adverse events (AEs), which include gastrointestinal AEs, headaches, weight loss and psychiatric symptoms [9, 12, 13].

BI 1015550 is an oral PDE4 inhibitor that preferentially inhibits PDE4B, and is a candidate drug for the treatment of IPF and other progressive fibrosing ILDs. Preclinical studies have demonstrated that BI 1015550 has anti-inflammatory and antifibrotic properties in *in vitro* and *in vivo* models of lung fibrosis [14]. *In vitro* findings include inhibition of human lung fibroblast proliferation and myofibroblast transformation, suggesting that BI 1015550 may have activity in patients with progressive fibrosing ILDs [14].

We describe the results from two early-phase clinical studies of BI 1015550. The first was a phase I study that aimed to investigate the safety, tolerability and pharmacokinetics of BI 1015550 in healthy male subjects. Based on the results from this study, a phase Ic study aimed to investigate the safety, tolerability and pharmacokinetics of BI 1015550 in male and female patients with IPF.

## Materials and methods

### Phase I study in healthy males

#### Study design

This phase I study (NCT03230487) was conducted between 15 August 2017 (first informed consent) and 16 January 2018 (study completion date of last subject) at CRS Clinical Research Services Mannheim GmbH, Mannheim, Germany. The independent ethics committee and competent authority approved the study. Written informed consent was obtained from all subjects prior to admission to the study.

Healthy males aged 18–45 years with a body mass index of 18.5–29.9 kg·m^−2^ were enrolled. Full inclusion and exclusion criteria are detailed in the supplementary methods.

Subjects received oral BI 1015550 or matching placebo in single rising doses (SRDs) of 36 mg and 48 mg, or multiple rising doses (MRDs) of 6 mg and 12 mg twice daily over 14 days. Both the SRD and MRD parts had a partially randomised, parallel-group design where the first block of each dose group was treated in a fixed sequence, whereas the second block was randomised in a 2/1 ratio. In the MRD part, subjects and investigators were both blinded to treatment allocation, whereas in the SRD part, only patients were blinded.

The SRD part was conducted under fasted conditions and the MRD part under fed conditions. In the MRD part, subjects were treated over 14 days and received a single morning dose on day 1, followed by 11 days of treatment (*i.e.* 6 mg twice daily, 12 mg twice daily or matching placebo on days 3 to 13), and a single morning dose on day 14. No treatments were administered on day 2 to allow 34-h pharmacokinetic sampling after a single dose.

Details of randomisation and blinding, subject allocation and sample size determination can be found in the supplementary methods.

#### Assessment endpoints

The primary endpoint was the number of subjects with drug-related AEs, with AEs graded as “mild” (awareness of signs or symptoms that were easily tolerated), “moderate” (sufficient discomfort to cause interference with usual activity) or “severe” (incapacitating or causing inability to work or to perform usual activities).

Pharmacokinetic parameters were analysed as secondary endpoints and are described in detail in the supplementary methods. Briefly, these included the peak plasma concentration (*C*_max_), the area under the concentration–time curve (AUC) from time zero to infinity (AUC_0−∞_) (SRD part) and accumulation ratios. In the MRD part, alongside *C*_max_, AUC was evaluated over a uniform dosing interval *τ* after the first dose (AUC*_τ_*_,1_) and over the dosing interval *τ* at steady state after the last dose (*C*_max,ss_ and AUC*_τ_*_,ss_).

Secondary safety endpoints included electrocardiogram (ECG), laboratory investigations and, in the MRD part, suicidality assessment; further details can be found in the supplementary methods. Descriptive statistics were calculated for all endpoints. No formal interim analysis was planned or performed.

### Phase Ic study in patients with IPF

#### Study design

This phase Ic study (www.clinicaltrials.gov identifier number NCT03422068) was conducted between 23 April 2018 (first informed consent) and 10 July 2019 (last patient visit) at 11 sites in seven European countries (supplementary table 1). Independent ethics committee approval from the participating centres was obtained prior to study initiation. Written informed consent was obtained from all patients prior to study admission.

**TABLE 1:** Phase I study in healthy male subjects: summary of adverse events (AEs)

	**SRD**				**MRD**			
	**Placebo**	**BI 1015550**			**Placebo**	**BI 1015550**		
		**36 mg**	**48 mg**	**Total**		**6 mg twice daily**	**12 mg twice daily**	**Total**
**Number of subjects**	6 (100.0)	6 (100.0)	6 (100.0)	12 (100.0)	8 (100.0)	8 (100.0)	8 (100.0)	16 (100.0)
**Subjects with any AE**	1 (16.7)	4 (66.7)	4 (66.7)	8 (66.7)	3 (37.5)	2 (25.0)	5 (62.5)	7 (43.8)
**Subjects with investigator-defined drug-related AEs**	1 (16.7)	2 (33.3)	4 (66.7)	6 (50.0)	1 (12.5)	2 (25.0)	4 (50.0)	6 (37.5)

Data are presented as n (%). SRD: single rising dose; MRD: multiple rising dose.

This study was conducted according to a randomised, double-blind, placebo-controlled, within-dose-groups design. Male and female patients with a diagnosis of IPF based on international guidelines [15], aged ≥40 years, who had not been treated with nintedanib or pirfenidone within 30 days of visit 1 and were not planning to be initiated on nintedanib or pirfenidone for the duration of the study were eligible. Full inclusion and exclusion criteria are detailed in the supplementary methods.

**FIGURE 1:**
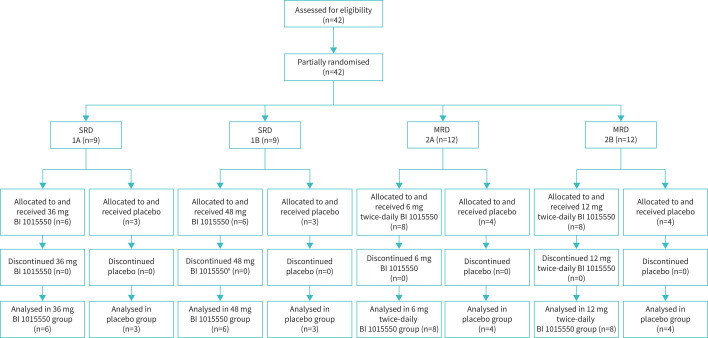
Phase I study in healthy males: subject flow. ^#^: one patient prematurely discontinued the study after taking 48 mg BI 1015550 due to an adverse event that was not considered drug-related (ligament sprain). SRD: single rising dose; MRD: multiple rising dose.

Two sequential doses were planned to be tested: 18 mg twice daily and 24 mg twice daily; however, dose escalation was stopped after the 18 mg twice-daily dose because exposure predictions for the 24 mg twice-daily dose group exceeded the predefined exposure threshold (supplementary methods). Due to challenges in recruitment, the duration of treatment was reduced from 12 weeks to 4 weeks, with patients recruited before this amendment treated up to a maximum of 12 weeks (figure 1).

Further details on the randomisation, blinding and allocation, determination of sample size and modifications to the study design can be found in the supplementary methods.

#### Assessment endpoints

The primary endpoint was the number of patients with drug-related AEs, with the severity grading the same as for the phase I study in healthy males. Pharmacokinetic parameters of BI 1015550 were evaluated as secondary endpoints after the first dose on day 1 (AUC*_τ_*_,1_ and *C*_max_) and after the morning dose on day 14 (AUC*_τ_*_,ss_ and *C*_max,ss_). Secondary safety endpoints included ECG, laboratory investigations and suicidality assessment; further details are provided in the supplementary methods. Exploratory lung function efficacy endpoints were changes in forced vital capacity (FVC), diffusing capacity of the lung for carbon monoxide and forced expiratory volume in 1 s. Descriptive statistics were calculated for all endpoints. No formal interim analysis was performed.

## Results

### Phase I study in healthy males

#### Subjects

The flow of subjects is shown in figure 1 and subject demographics in supplementary table 2. Demographic and baseline characteristics were similar between the treatment groups.

**TABLE 2:** Phase I study in healthy male subjects: summary of pharmacokinetic parameters of BI 1015550 in the single rising dose part

**Parameter (unit)**	**BI 1015550 36 mg^#^**		**BI 1015550 48 mg^#^**	
	**gMean**	**gCV (%)**	**gMean**	**gCV (%)**
***C*_max_ (nmol·L^−1^)**	710	20.7	955	15.5
**AUC_0−∞_ (nmol·h·L^−1^)**	5910	21.2	8700^¶^	17.2
**fe_0–120_ (%)**	12.5^¶^	45.2	12.1^+^	13.4
**CL_R,0–120_ (mL·min^−1^)**	30.5^+^	21.5	25.2^+^	20.6

gMean: geometric mean; gCV: geometric coefficient of variation; *C*_max­_: maximum measured concentration of analyte in plasma; AUC_0–__∞_: area under the concentration–time curve of the analyte in plasma over the time interval from 0 extrapolated to ∞; fe_0–120_: fraction of administered drug excreted unchanged in urine over the time interval 0–120** **h after first drug administration; CL_R,0–120_: renal clearance of the analyte in plasma over the time interval 0–120** **h after first drug administration. ^#^: fasted, n=6; ^¶^: n=5; ^+^: n=4.

#### Safety

A summary of AEs is shown in table 1, all AEs are shown in supplementary table 3 and salient laboratory parameters are shown in supplementary table 4.

**TABLE 3:** Phase I study in healthy male subjects: summary of pharmacokinetic parameters of BI 1015550 in the multiple rising dose part

**Parameter (unit)**	**BI 1015550 6 mg twice daily^#^**		**BI 1015550 12 mg twice daily^#^**	
	**gMean**	**gCV (%)**	**gMean**	**gCV (%)**
***C*_max_ (nmol·L^−1^)**	103	28.2	229	29.9
**AUC*_τ_*_,1_ (nmol·h·L^−1^)**	564	24.8	1370	15.9
***C*_max,ss_ (nmol·L^−1^)**	164	21.3	348	14.1
**AUC*_τ_*_,ss_ (nmol·h·L^−1^)**	1050	25.7	2300	15.8
***R*_A,*C*_max__**	1.60	35.0	1.52	23.6
***R*_A,AUC_**	1.85	9.91	1.68	14.8

gMean: geometric mean; gCV: geometric coefficient of variation; *C*_max­_: maximum measured concentration of analyte in plasma; AUC*_τ_*_,1_: area under the concentration–time curve for the analyte in plasma over a uniform dosing interval *τ* after the first dose; *C*_max,ss_: maximum measured concentration of analyte in plasma at steady state over a uniform dosing interval *τ*; AUC*_τ_*_,ss_: area under the concentration–time curve of the analyte in plasma at steady state over a uniform dosing interval *τ*; *R*_A,*C*_max__: accumulation ratio of the analyte in plasma after multiple dose administration over a uniform dosing interval *τ*, expressed as a ratio of *C*_max_ at steady state and after a single dose; *R*_A,AUC_: accumulation ratio of the analyte in plasma after multiple dose administration over a uniform dosing interval *τ*, expressed as a ratio of AUC at steady state and after a single dose. ^#^: fed, n=8.

**TABLE 4:** Phase Ic study in patients with idiopathic pulmonary fibrosis: summary of adverse events (AEs)

	**Placebo**	**BI 1015550** **18 mg twice daily**	**Total on treatment**
**Patients treated**	5 (100.0)	10 (100.0)	15 (100.0)
**Any AE**	5 (100.0)	10 (100.0)	15 (100.0)
**Severe AEs**	0 (0.0)	1 (10.0)	1 (6.7)
**Investigator-defined drug-related AE**	3 (60.0)	9 (90.0)	12 (80.0)
**AE leading to discontinuation of study drug**	0 (0.0)	1 (10.0)	1 (6.7)
**Patients with AESI^#^**	0 (0.0)	0 (0.0)	0 (0.0)
**Patients with other significant AEs according to ICH E3**	0 (0.0)	1 (10.0)	1 (6.7)
**Patients with SAEs**	0 (0.0)	1 (10.0)	1 (6.7)
Patients requiring or prolonging hospitalisation	0 (0.0)	1 (10.0)	1 (6.7)

Data are presented as n (%). AESI: adverse event of special interest; ICH: International Council for Harmonisation of Technical Requirements for Pharmaceuticals for Human Use; SAE: serious adverse event. ^#^: hepatic injury was defined as an AESI.

AEs were reported more frequently for patients treated with BI 1015550 *versus* placebo in the SRD part (66.7% *versus* 16.7%), and with similar frequencies in the MRD part (43.8% *versus* 37.5%).

In the SRD part, the most common AEs by organ class were nervous system disorders (headache and dizziness), reported for 41.7% of patients receiving BI 1015550 and 16.7% of patients treated with placebo. This was largely driven by headache as there was only one case of dizziness (36 mg SRD part) in the whole study. The second most common AEs by organ class were gastrointestinal disorders (abdominal distension, upper abdominal pain, constipation, diarrhoea and nausea), reported for 25.0% of patients receiving BI 1015550 and 16.7% of patients receiving placebo.

In the MRD part, the most common AEs by organ class were nervous system disorders (headache), reported for 31.3% of patients receiving BI 1015550 and 12.5% of patients treated with placebo. The second-most common AEs by organ class were gastrointestinal disorders (abdominal distension, diarrhoea, nausea and oral hypoesthesia), reported for 18.8% of patients receiving BI 1015550 and 12.5% of patients receiving placebo.

All AEs were mild or moderate in intensity and resolved before the end of the study.

One subject in the 48 mg treatment group prematurely discontinued study participation after a single dose due to an AE (ligament sprain) that was not considered drug-related.

There were no reported deaths, severe AEs, serious AEs, protocol-specified AEs of special interest or other significant AEs (according to International Council for Harmonisation of Technical Requirements for Pharmaceuticals for Human Use E3), and no clinically relevant findings with respect to ECG recordings or vital signs. The only clinically relevant laboratory finding was an increase in blood triglycerides in one subject in the 12 mg twice-daily treatment group, which was considered drug-related. No incidence of suicidal ideation or behaviour were detected using the Columbia Suicide Severity Rating Scale (C-SSRS).

Body weight was measured in the MRD part, where there was a trend for a decrease in weight in the BI 1015550 treatment groups only. In the 6 mg and 12 mg twice-daily treatment groups, the mean±sd changes from baseline were –1.21±0.43 and –1.34±1.61 kg, respectively. In the placebo MRD group, the mean change from baseline was 0.21±1.06 kg.

#### Pharmacokinetics

**FIGURE 2:**
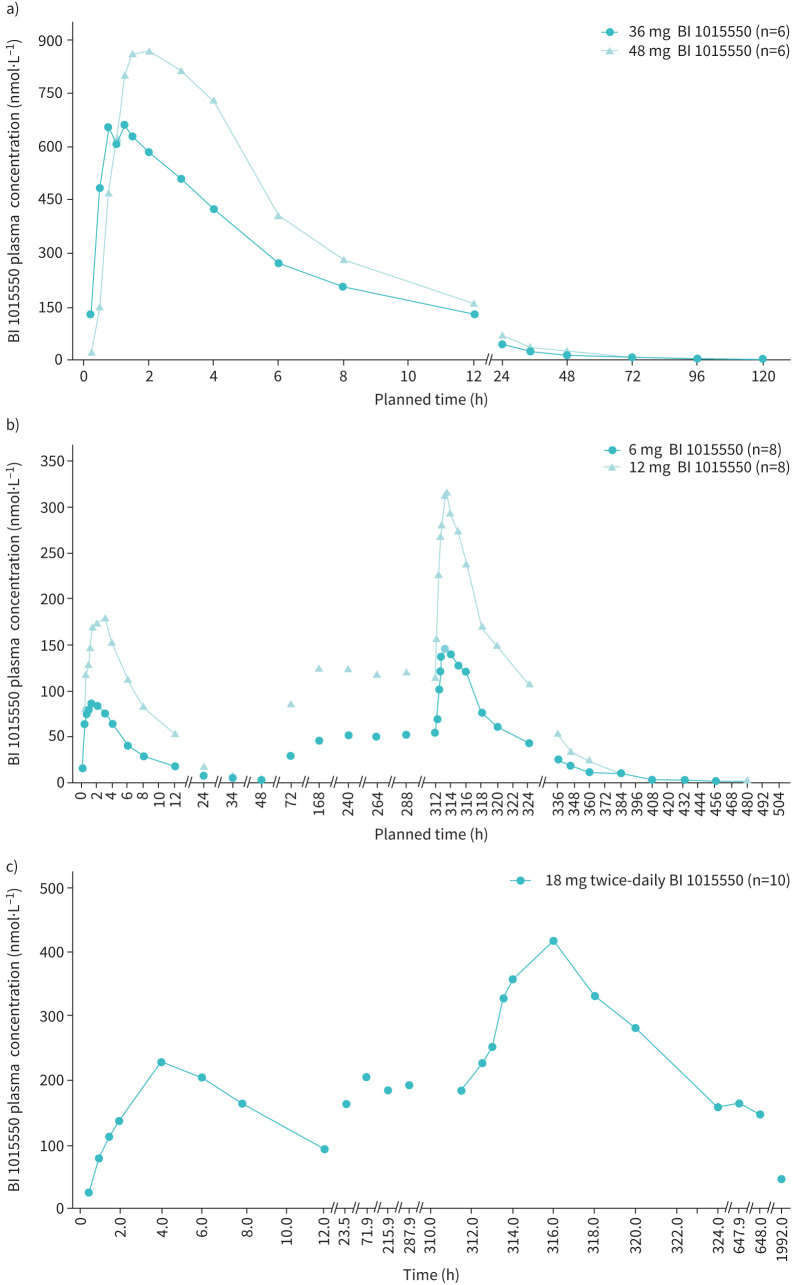
Geometric mean plasma concentration–time profiles of BI 1015550. Phase I study in healthy males after a) single oral administration of BI 1015550 36 mg or 48 mg under fasted conditions, and b) single and multiple oral administrations of 6 mg or 12 mg twice-daily BI 1015550 under fed conditions. c) Phase Ic study in patients with idiopathic pulmonary fibrosis after single and multiple oral administration of 18 mg twice-daily BI 1015550.

Pharmacokinetic parameters are shown in tables 2 and 3. The geometric mean (gMean) plasma concentration–time profiles after single and multiple doses are shown in figure 2. Plasma concentrations increased quickly. They reached a peak concentration of the analyte in plasma with a median time from last dosing to the maximum measured concentration of 1.25–1.52 h after single and multiple oral administration, and then declined with terminal half-lives of 16–27 h.

Linear pharmacokinetics with a dose-proportional increase in AUC of the analyte in plasma were observed for the dose ranges tested (from 36 mg to 48 mg single dose and from 6 mg to 12 mg twice-daily BI 1015550). A steady state was reached by day 6, with a slight accumulation after multiple twice-daily administrations. gMean accumulation ratios based on *C*_max_ were 1.60 and 1.52 for 6 mg twice daily and 12 mg twice daily, respectively. gMean accumulation ratios based on AUC*_τ_*_,1_ were 1.85 and 1.68 for 6 mg twice daily and 12 mg twice daily, respectively.

### Phase Ic study in patients with IPF

#### Patients

**FIGURE 3:**
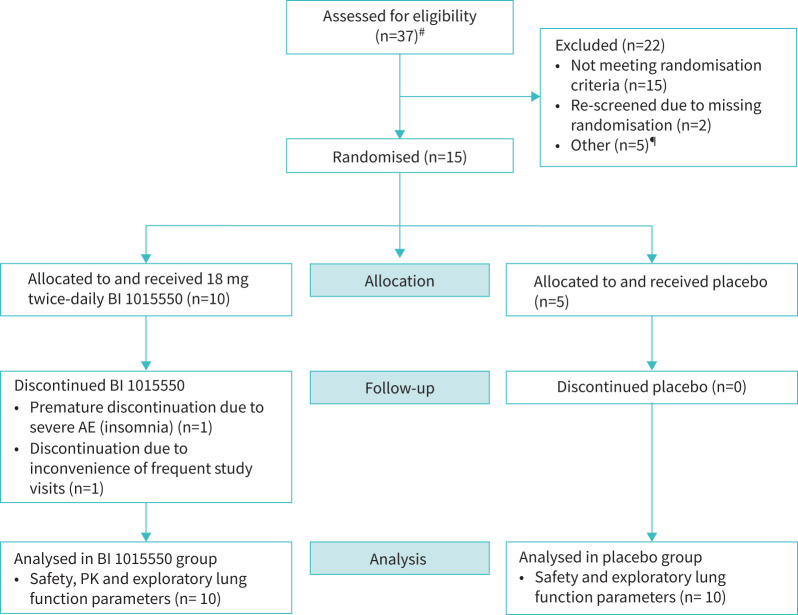
Phase Ic study in patients with idiopathic pulmonary fibrosis: patient flow. Of 10 patients treated with BI 1015550, seven were treated up to a maximum duration of 12 weeks and three up to a maximum of 4 weeks. Of five patients treated with placebo, four were treated up to a maximum duration of 12 weeks and one up to a maximum of 4 weeks. AE: adverse event; PK: pharmacokinetic. ^#^: four patients were screened twice; ^¶^: other reasons for discontinuations (n=1 each) included no longer willing to participate, administrative reason, randomisation timeline, study closed and unable to perform calprotectin retest.

The flow of patients with IPF is shown in figure 3 and patient demographics in supplementary table 4. Ten patients were treated with BI 1015550 and five patients with placebo for a median duration of 53.5 and 84.0 days, respectively. Baseline lung function was comparable between the treatment groups.

#### Safety

A summary of AEs is shown in table 4, all AEs are shown in supplementary table 6 and salient laboratory parameters are shown in supplementary table 7.

The most frequently reported AEs by organ class were gastrointestinal disorders, reported in eight (80.0%) patients treated with BI 1015550 and two (40.0%) patients receiving placebo, followed by infections and infestations, reported in five (50.0%) patients treated with BI 1015550 and two (40.0%) patients receiving placebo. The most frequent infection was nasopharyngitis, reported in four (40%) patients treated with BI 1015550. The most frequent gastrointestinal events were diarrhoea, which affected four (40.0%) patients receiving BI 1015550 and placebo. All patients with diarrhoea had recovered by the end of the trial.

There was one severe AE of insomnia in a patient treated with BI 1015550, which was considered drug related and stopped when the patient discontinued BI 1015550 treatment. This was the only severe AE in the study and the only AE leading to discontinuation. All other AEs were mild or moderate in intensity and most were resolved by the end of the study. There were no reported deaths, and no cases of suicidal ideation or behaviour were detected using the C-SSRS.

One patient in the BI 1015550 group experienced two serious AEs (SAEs) (anal fistula and anal incontinence) that were mild in intensity. This patient had a long history of anal fistula, and was scheduled for an elective surgical treatment of anal fistula and secondary anal incontinence. Anal fistula and anal incontinence were categorised as SAEs due to hospitalisation but were not considered drug-related.

There were no clinically relevant findings with respect to 12-lead ECG or vital signs. Laboratory tests also revealed no clinically relevant findings except for a slight increase in C-reactive protein in the BI 1015550 group.

Overall, there was no notable difference between the two treatment groups with respect to body weight. Mean observed weight loss in patients treated for up to 12 weeks was –1.18 kg in the placebo group and –1.74 kg in the BI 1015550 group, and this small numerical difference was not consistent over time.

#### Pharmacokinetics

A summary of the pharmacokinetic parameter results is shown in table 5. gMean plasma concentration–time profiles after single and multiple doses of BI 1015550 18 mg twice daily are shown in figure 2. The gMeans for *C*_max_ and AUC*_τ_*_,1_ were higher after multiple administrations than after administration of a single dose. The gMean for accumulation ratios based on *C*_max_ and AUC*_τ_* was 1.66 and 1.87, respectively. Interindividual variability was generally low to moderate after administration of a single dose, and moderate to high after multiple administrations. After approximately five administrations of BI 1015550 18 mg twice daily, 95% of the steady-state concentration was reached.

**TABLE 5:** Phase Ic study in patients with idiopathic pulmonary fibrosis: pharmacokinetic parameters of BI 1015550 18 mg twice daily

**Parameter (unit)**	**gMean^+^**	**gCV (%)**
**Day 1^#^**		
*C*_max_ (nmol·L^−1^)	277	23.1
AUC*_τ_*_,1_ (nmol·h·L^−1^)	1990	18.2
**Day 14^¶^**		
*C*_max,ss_ (nmol·L^−1^)	460	41.7
AUC*_τ_*_,ss_ (nmol·h·L^−1^)	3720	49.5

n=10. gMean: geometric mean; gCV: geometric coefficient of variation; *C*max: maximum measured concentration of the analyte in plasma; AUC*_τ_*_,1_: area under the concentration–time curve of the analyte in plasma over a uniform dosing interval τ after administration of the first dose; *C*_max,ss_: maximum measured concentration of analyte in plasma at steady state over a uniform dosing interval *τ*; AUC*τ*,ss: area under the concentration–time curve of the analyte in plasma at steady state over a uniform dosing interval τ at steady state. ^#^: after the first dose; ^¶^: at steady state; ^+^: n=10.

#### Exploratory efficacy endpoints

Lung function parameter data were highly variable between patients and no clear effect of BI 1015550 treatment on lung function parameters could be observed over the course of the trial. There was a trend towards a slight reduction in FVC over time in the placebo group; this trend was not observed in the BI 1015550 group (data not shown).

## Discussion

The results of the phase I study show that BI 1015550 has an acceptable safety and tolerability profile in healthy male subjects. Overall, the total exposure to BI 1015550 appeared to increase proportionally with dose over the range tested. In the phase Ic study, only the 18 mg twice-daily dose was investigated because the pharmacokinetic exposure prediction for the 24 mg twice-daily dose exceeded the predefined exposure threshold. In the phase Ic study, BI 1015550 at 18 mg twice daily had acceptable safety and tolerability in patients with IPF who had not received background antifibrotic treatment. After approximately five administrations at 18 mg twice daily, 95% of the steady-state concentration was reached. There was no difference in pharmacokinetic parameters between healthy volunteers and patients with IPF.

The use of oral PDE4 inhibitors is currently limited due to their association with AEs such as gastrointestinal AEs and headache [9]. An alternative therapeutic strategy to reduce the AEs associated with oral PDE4 inhibitors is to inhibit PDE4B preferentially, potentially leading to anti-inflammatory and antifibrotic effects whilst circumventing many of the AEs associated with more general PDE4 inhibitors [9, 16, 17].

In the phase Ic and I studies, gastrointestinal disorders were the first- and second-most commonly reported adverse events by organ class, respectively. In the phase I study, the most common AEs by system organ class were nervous system disorders, driven by headache. Both gastrointestinal disorders and headache are known class effects of other nonselective PDE4 inhibitors [9]. In both trials, suicidal ideation and behaviour and weight loss were monitored because they are listed as side effects associated with marketed oral PDE4 inhibitors [12]. No suicidal ideation or behaviour was reported in either of our studies. In the phase I trial in healthy volunteers, there was a slight trend for a decrease in weight in subjects treated with BI 1015550, but there were no notable differences between treatment groups in the phase Ic trial in patients with IPF.

BI 1015550, an oral preferential inhibitor of PDE4B, is the first PDE4 inhibitor to be investigated in patients with IPF. Preclinical data have shown that BI 1015550 has anti-inflammatory and antifibrotic effects [14]. BI 1015550 also appears to have a complementary mode of action with nintedanib on fibroblast transformation and synergistic effects on fibroblast proliferation [14]. A limitation of the phase Ic study is that recruitment was restricted to patients who were not receiving background antifibrotic treatment. This precluded investigation of potential additive effects on the efficacy and/or safety of BI 1015550 in combination with background antifibrotic standard of care. Such effects are, however, being investigated in a phase II study of BI 1015550 in patients with IPF with and without background antifibrotic treatment, which has recently completed (www.clinicaltrials.gov identifier number NCT04419506) [18].

Further limitations of both studies include the small sample size, lack of diversity among study participants and short study duration. Potential effects of sex, race and/or ethnicity on bioavailability and clearance of BI 1015550 will be examined in future studies in more diverse populations. In the phase I study, there was possible observer bias of dose-dependent and time-dependent effects. In the phase Ic study, patients with IPF had relatively preserved lung function (mean FVC 91.7% predicted); therefore, the effects in patients with more severe disease are unknown.

In conclusion, the results of these studies suggest that BI 1015550 has an acceptable safety and tolerability profile in healthy male subjects and in male and female patients with IPF within the dose range tested and for up to 12 weeks. There were no obvious differences in pharmacokinetic parameters between healthy volunteers and patients with IPF. These data support further clinical studies to investigate the safety and efficacy of BI 1015550 18 mg twice daily in larger and more diverse populations of patients with IPF.

## Supplementary material

10.1183/23120541.00240-2022.Supp1 **Please note:** supplementary material is not edited by the Editorial Office, and is uploaded as it has been supplied by the author.
Supplementary material 00240-2022.SUPPLEMENT
