# Supplementary material for: Phase I studies of BI 1015550, a preferential phosphodiesterase 4B inhibitor, in healthy males and patients with idiopathic pulmonary fibrosis
Source: ERJ Open Res. 2022 Oct 24;8(4):00240-2022. doi: 10.1183/23120541.00240-2022 (PMC9589333; doi:10.1183/23120541.00240-2022)
Supplement: Supplementary file 1 [file 00240-2022.SUPPLEMENT.pdf]

Supplementary Material

Phase I studies of BI 1015550, a preferential PDE4B inhibitor, in healthy males and patients with idiopathic pulmonary fibrosis

Toby M. Maher, Christina Schlecker, Doreen Luedtke, Sebastian Bossert, Donald F. Zoz, Armin Schultz

Supplementary Methods: Phase I study in healthy males

Inclusion criteria

1. Healthy male subjects according to the investigator's assessment, based on a complete medical history including a physical examination, vital signs, 12-lead electrocardiogram (ECG) and clinical laboratory tests.
2. Age of 18 to 45 years (inclusive).
3. Body mass index of 18.5 to 29.9 kg/m^2 (inclusive).

Main exclusion criteria

1. Any finding in the medical examination (including vital signs or ECG) that deviated from normal and was judged as clinically relevant by the investigator.
2. Repeated measurement of systolic blood pressure outside the range of 90 to 140 mmHg, diastolic blood pressure outside the range of 55 to 90 mmHg, or pulse rate outside the range of 50 to 90 bpm.
3. Any laboratory value outside the reference range that the investigator considered to be of clinical relevance.
4. Any evidence of a concomitant disease judged as clinically relevant by the investigator.
5. Gastrointestinal, hepatic, renal, respiratory, cardiovascular, metabolic, immunological or hormonal disorders.

6. Cholecystectomy and/or surgery of the gastrointestinal tract that could interfere with the pharmacokinetics of the study medication (except appendectomy).
7. Diseases of the central nervous system (including but not limited to any kind of seizures or stroke), and other relevant neurological or psychiatric disorders, including but not limited to mood disorders and any history of suicidality.
8. History of relevant orthostatic hypotension, fainting spells or blackouts.
9. Chronic or relevant acute infections.
10. History of relevant allergy or hypersensitivity (including allergy to the study medication or its excipients).
11. Any lifetime history of suicidal behaviour (i.e. actual attempt, interrupted attempt, aborted attempt, or preparatory acts or behaviour).
12. Any suicidal ideation (i.e. type 1 to 5) on the Columbia Suicide Severity Rating Scale (C-SSRS) in the 12 months preceding screening (i.e. passive/active suicidal thought, active suicidal thought with method, active suicidal thought with intent but without specific plan, or active suicidal thought with plan and intent; only in the multiple-rising-dose [MRD] part).

Randomisation, blinding and allocation

Subjects were partially randomised within each dose group. The first block of each dose group was treated in a fixed sequence (BI 1015550 – placebo – BI 1015550), while the other block was randomised in a 2:1 ratio reflecting the ratio of subjects receiving BI 1015550 to placebo.

Randomisation was arranged by the sponsor Boehringer Ingelheim Pharma GmbH & Co. KG, Biberach an der Riss, Germany. The randomisation list was generated using a validated system, which involved a pseudo-random number generator and a supplied seed number so that the resulting allocation was both reproducible and non-predictable. Block size was 3.

In the single-rising-dose (SRD) part (1A and 1B), the treatments administered (BI 1015550 or placebo) were single-blind (blinded to subjects only); however, the current dose level was known to subjects. Subjects received a single dose of treatment. In the MRD part (2A and 2B), the treatments administered were double-blind to subjects, investigators and research staff at the study site; however, the current dose level was known to subjects and investigators.

Subjects were recruited to dose groups according to their temporal availability. As soon as enough subjects had been allocated to one of the two dose cohorts (two cohorts per dose group), subjects were allocated to one of the other dose cohorts. Therefore, the allocation of subjects to dose cohorts or groups was not influenced by study personnel, but only by the subjects' temporal availability.

Secondary endpoints

Pharmacokinetic parameters were analysed as secondary endpoints for BI 1015550: for the SRD part, these were area under the concentration−time curve (AUC) of the analyte in plasma over the time interval from 0 extrapolated to infinity ($AUC_{0-\infty}$) and maximum measured concentration of the analyte in plasma (C_{max}); for the MRD part, AUC of the analyte in plasma over a uniform dosing interval τ after the first dose ($AUC_{\tau,1}$) and C_{max} were evaluated after the first dose. AUC of the analyte in plasma over the dosing interval τ at steady state ($AUC_{\tau,ss}$) and C_{max} at steady state over a uniform dosing interval τ ($C_{max,ss}$) were evaluated after the last dose. The following accumulation ratios were analysed: analyte in plasma after multiple dose administration over a uniform dosing interval τ, expressed as ratio of AUC at steady state and after single dose ($R_{A,AUC}$) and analyte in plasma after multiple dose administrations over a uniform dosing interval τ, expressed as ratio of C_{max} at steady state and after single dose ($R_{A,Cmax}$). Further pharmacokinetic parameters that were analysed were renal clearance of the analyte in plasma over the time interval 0 to 120 hours after first drug administration ($CL_{R,0-120}$) and fraction of administered drug excreted unchanged in urine over the time interval from 0 to 120 hours after first drug administration (fe_{0-120}). BI 1015550

concentrations in plasma and urine were determined using high-performance liquid chromatography with tandem mass spectrometry (HPLC-MS).

Secondary safety endpoints were treatment-emergent adverse events (TEAEs) including clinically relevant findings from the physical examination, 12-lead ECG, continuous ECG monitoring (SRD part only), vital signs (blood pressure, pulse rate, respiratory rate, aural body temperature), body weight (MRD part only) suicidality assessment (using the C-SSRS; MRD part only), and safety tests for the following functional laboratory groups: haematology, differentials, coagulation, enzymes, hormones, substrates, and electrolytes, as well as tests for faecal occult blood, faecal calprotectin and urinalysis for haematuria..

Determination of sample size

A total of 42 subjects were planned to be included in this study. The planned sample sizes were not based on a power calculation.

Supplementary methods: Phase Ic study in patients with idiopathic pulmonary fibrosis (IPF)

Inclusion criteria

1. Male or female patients aged ≥40 years at Visit 1.
2. A clinical diagnosis of IPF based on the American Thoracic Society/European Respiratory Society/Japanese Respiratory Society/Latin American Thoracic Association 2011 Guidelines (P11-07084) within the previous 5 years as confirmed by the investigator based on a chest high-resolution computed tomography scan taken within 12 months of Visit 1, with a pattern of usual interstitial pneumonia confirmed by central review prior to Visit 2.
3. Forced vital capacity (FVC) ≥50% of predicted normal at Visit 1.
4. Diffusing capacity of the lung for carbon monoxide (corrected for haemoglobin [Visit 1]): >30% of predicted normal at Visit 1.

Main exclusion criteria

1. Patients with a significant disease or condition other than IPF, which in the opinion of the investigator may have put the patient at risk due to participation, may have interfered with study procedures, or may have caused concern regarding the patient's ability to participate in the study.
2. Any laboratory value outside the reference range that the investigator considered to be of clinical relevance.
3. Surgery of the gastrointestinal tract that could have interfered with the pharmacokinetics of the study medication (except appendectomy).
4. Diseases of the central nervous system (including but not limited to any kind of seizures or stroke) and other relevant neurological or psychiatric disorders (including but not limited to mood disorders).
5. Evidence of active infection (chronic or acute) based on clinical examination or laboratory findings.
6. History of allergy or hypersensitivity to the study medication or its excipients.
7. Relevant airway obstruction (i.e. prebronchodilator forced expiratory volume in 1 second/ FVC <0.7) at Visit 1.
8. Patients who had previously been treated with nintedanib or pirfenidone within 30 days of Visit 1.
9. Positive faecal occult blood (no retest allowed).
10. Positive testing for haematuria if confirmed by microscopic urine analysis (retest allowed).
11. Any lifetime history of suicidal behaviour (i.e. actual attempt, interrupted attempt, aborted attempt, or preparatory acts or behaviour).
12. Any suicidal ideation of type 2 to 5 on the C-SSRS in the past 12 months (i.e. active suicidal thought without method, intent or plan; active suicidal thought with method,

but without intent or plan; active suicidal thought with method and intent but without specific plan; or active suicidal thought with method, intent and plan).

Randomisation, blinding and allocation

Randomisation was performed by Boehringer Ingelheim Pharma GmbH & Co. KG, Biberach an der Riss, Germany. Randomisation was performed using an Interactive Response Technology, which was provided by PAREXEL International GmbH. The randomisation code was generated using a validated system, which involved a pseudo-random number generator and a supplied seed number so that the resulting allocation was both reproducible and non-predictable and verified by a trial-independent statistician.

This study was double-blind with regard to the patients, investigators and research staff at the study sites in order to eliminate observer or performance bias. According to the MRD study design, the dose level was known to patients and investigators.

Secondary endpoints

Pharmacokinetic parameters of BI 1015550 were evaluated as secondary endpoints after the first dose on Day 1 ($AUC_{\tau,1}$ and C_{max}) and after the morning dose on Day 14 ($AUC_{\tau,ss}$ and $C_{max,ss}$). BI 1015550 concentrations in plasma were determined using HPLC-MS.

Secondary safety endpoints were TEAEs including clinically relevant findings from the physical examination, 12-lead ECG, vital signs (blood pressure, pulse rate, respiratory rate, oral body temperature, suicidality monitoring (using the C-SSRS) and safety laboratory tests for the following functional groups: haematology, differentials, coagulation, enzymes, hormones, substrates, plasma proteins, inflammatory parameters, and electrolytes, as well as tests for faecal occult blood, faecal calprotectin and urinalysis for haematuria.

Determination of sample size

It was planned to include 18 patients to be allocated to two dose groups in this study, but only one dose group was tested (see Modifications to the study design).

Modifications to the study design

Two sequential BI 1015550 dose groups were planned that were within the estimated therapeutic range and were based on the safety profile of this class of compound (18 mg and 24 mg; twice daily [BID]). However, only the 18 mg BID dose group was evaluated because one of the predefined stopping criteria for dose escalation was met (exposure predictions area under the concentration-time curve of the analyte in plasma over the time interval from 0 to 24 h at steady state $[AUC_{0-24,ss}]$ for the 24 mg BID dose group exceeded the exposure threshold).

Originally, this study was to consist of a second study part, where BI 1015550 was planned to be evaluated on top of antifibrotic standard of care. However, due to recruitment challenges, and as this was the first investigation of the compound in MRD of 18 mg, as well as the first study in patients, the second study part was removed. This enabled a greater focus on safety in a more homogeneous group of patients with IPF not treated with background antifibrotics. Potential additive effects on the safety and/or efficacy of BI 1015550 in combination with background antifibrotic standard of care are being investigated in a Phase II study of BI 1015550, which has recently completed (NCT04419506).

Due to challenges in recruitment, duration of treatment was reduced from 12 weeks to 4 weeks. Patients who entered the study before approval of this amendment could be treated up to a maximum treatment duration of 12 weeks. These changes were implemented to help with recruitment and to keep the focus on the main objective of the trial, which was safety.

Additional patients were included based on the experience gained during the study (e.g. preliminary pharmacokinetic data). As the observed pharmacokinetic data of the first cohort showed higher variability in patients with IPF than in healthy volunteers, it was considered appropriate to extend the number of patients in the 18 mg BID group. Due to a protocol amendment, additional patients (four on active treatment and two on placebo) could be included in the 18 mg BID dose group.

Supplementary Table 1. Phase Ic study in patients with IPF: list of investigators for each study site

Principal Investigator	Site
I. Titlestad	Odense Universitetshospital<br>Lungemedicinsk Afdeling J,<br>Forskningsenheden<br>Odense<br>Denmark
M. Myllärniemi	HYKS Keuhkosairauksien tutkimusyksikkö Biomedicum Helsinki 2<br>Helsinki<br>Finland
M. Kilpeläinen	TYKS, Keuhkosairauksien klinikka,<br>T-sairaala<br>Turku<br>Finland
M. Kreuter	Universitätsklinikum Heidelberg<br>Zentrum für interstitielle & seltene<br>Lungenerkrankungen<br>Thoraxklinik<br>Heidelberg<br>Germany
A. Prasse	Fraunhofer Institut für Toxikologie und Experimentelle Medizin (ITEM)<br>Hannover<br>Germany
L. Richeldi	Policlinico Gemelli Unità Complessa<br>Pneumologia<br>Unità Complessa Pneumologia

	Rome<br>Italy
M. Wijsenbeek	Erasmus Medisch Centrum<br>Rotterdam<br>Netherlands
M. Veltkamp	St. Antonius Ziekenhuis, Locatie<br>Nieuwegein<br>R&D Lung Diseases<br>Nieuwegein<br>Netherlands
M. Molina-Molina	Hospital Universitari de Bellvitge<br>Servicio de Neumologia<br>L'Hospitalet de Llobregat<br>Barcelona<br>Spain
P. Molyneaux	Royal Brompton Hospital<br>Dept of Respiratory Medicine<br>Brompton & Harefield Hospital NHS<br>Trust<br>London<br>United Kingdom
S. Fletcher	Southampton General Hospital<br>Southampton Centre for Biomedical<br>Research, C Level West Wing,<br>University Hospital Southampton NHS<br>Foundation Trust<br>Southampton<br>United Kingdom

IPF, idiopathic pulmonary fibrosis.

Supplementary Table 2. Phase I study in healthy males: baseline demographics

Demographic	SRD			MRD			Total
	Placebo	BI 1015550 36 mg	BI 1015550 48 mg	Placebo	BI 1015550 6 mg BID	BI 1015550 12 mg BID	
**Number of subjects n (%)**	6 (100.0)	6 (100.0)	6 (100.0)	8 (100.0)	8 (100.0)	8 (100.0)	42 (100.0)
**Male n (%)**	6 (100.0)	6 (100.0)	6 (100.0)	8 (100.0)	8 (100.0)	8 (100.0)	42 (100.0)
**Age in years, mean (SD)**	34.3 (7.9)	32.2 (6.6)	38.2 (7.9)	33.8 (6.6)	35.8 (8.7)	33.5 (5.3)	34.6 (7.0)
**Height in cm, mean (SD)**	174.2 (4.9)	180.7 (7.9)	175.8 (5.4)	174.8 (7.9)	176.9 (6.3)	177.4 (4.2)	176.6 (6.2)
**Weight in kg, mean (SD)**	76.5 (11.1)	79.3 (6.9)	79.3 (9.4)	70.7 (9.2)	79.2 (9.5)	83.5 (8.4)	78.0 (9.5)
**BMI in kg/m^2, mean (SD)**	25.2 (3.8)	24.3 (2.0)	25.6 (2.5)	23.1 (2.4)	25.4 (3.2)	26.5 (1.6)	25.0 (2.7)
**Race, n (%)[#]**							
White	6 (100.0)	5 (83.3)	6 (100.0)	6 (75.0)	8 (100.0)	8 (100.0)	39 (92.9)
Black or African American	0 (0.0)	1 (12.5)	0 (0.0)	1 (12.5)	0 (0.0)	0 (0.0)	2 (4.8)
Asian	0 (0.0)	0 (0.0)	0 (0.0)	1 (12.5)	0 (0.0)	0 (0.0)	1 (2.4)
**Smoking history n (%)**							
Never smoked	2 (33.3)	2 (33.3)	4 (66.7)	5 (62.5)	2 (25.0)	3 (37.5)	18 (42.9)
Former smoker	3 (50.0)	2 (33.3)	1 (16.7)	1 (12.5)	3 (37.5)	2 (25.0)	12 (28.6)
Currently smokes	1 (16.7)	2 (33.3)	1 (16.7)	2 (25.0)	3 (37.5)	3 (37.5)	12 (28.6)

**Alcohol history n (%)**							
Non-drinker	1 (16.7)	0 (0.0)	1 (16.7)	2 (25.0)	2 (25.0)	2 (25.0)	8 (19.0)
Drinks – no interference	5 (83.3)	6 (100.0)	5 (83.3)	6 (75.0)	6 (75.0)	6 (75.0)	34 (81.0)
Drinks – possible interference	0 (0.0)	0 (0.0)	0 (0.0)	0 (0.0)	0 (0.0)	0 (0.0)	0 (0.0)

[#]None of the subjects were Hispanic/Latino. BID, twice daily; BMI, body mass index; MRD, multiple-rising-dose; SD, standard deviation; SRD, single-rising-dose.

Supplementary Table 3. Phase I study in healthy males: all AEs

System organ class, preferred term	SRD				MRD			
	Placebo n (%)	BI 1015550 36 mg n (%)	BI 1015550 48 mg n (%)	BI 1015550 Total n (%)	Placebo n (%)	BI 1015550 6 mg BID n (%)	BI 1015550 12 mg BID n (%)	BI 1015550 Total n (%)
**Number of subjects**	6 (100.0)	6 (100.0)	6 (100.0)	12 (100.0)	8 (100.0)	8 (100.0)	8 (100.0)	16 (100.0)
**Total with AEs**	1 (16.7)	4 (66.7)	4 (66.7)	8 (66.7)	3 (37.5)	2 (25.0)	5 (62.5)	7 (43.8)
**Nervous system disorders**	1 (16.7)	2 (33.3)	3 (50.0)	5 (41.7)	1 (12.5)	2 (25.0)	3 (37.5)	5 (31.3)
Headache	1 (16.7)	1 (16.7)	3 (50.0)	4 (33.3)	1 (12.5)	2 (25.0)	3 (37.5)	5 (31.3)
Dizziness	0 (0.0)	1 (16.7)	0 (0.0)	1 (8.3)	0 (0.0)	0 (0.0)	0 (0.0)	0 (0.0)
**Gastrointestinal disorders**	1 (16.7)	2 (33.3)	1 (16.7)	3 (25.0)	1 (12.5)	2 (25.0)	1 (12.5)	3 (18.8)
Abdominal distension	0 (0.0)	0 (0.0)	1 (16.7)	1 (8.3)	1 (12.5)	0 (0.0)	0 (0.0)	0 (0.0)
Upper abdominal pain	0 (0.0)	1 (16.7)	0 (0.0)	1 (8.3)	0 (0.0)	0 (0.0)	0 (0.0)	0 (0.0)
Constipation	0 (0.0)	1 (16.7)	0 (0.0)	1 (8.3)	0 (0.0)	0 (0.0)	0 (0.0)	0 (0.0)
Diarrhoea	0 (0.0)	1 (16.7)	0 (0.0)	1 (8.3)	1 (12.5)	1 (12.5)	0 (0.0)	1 (6.3)
Nausea	1 (16.7)	0 (0.0)	0 (0.0)	0 (0.0)	0 (0.0)	1 (12.5)	0 (0.0)	1 (6.3)
Oral hypoaesthesia	0 (0.0)	0 (0.0)	0 (0.0)	0 (0.0)	0 (0.0)	0 (0.0)	1 (12.5)	1 (6.3)

**Investigations**	0 (0.0)	1 (16.7)	0 (0.0)	1 (8.3)	1 (12.5)	0 (0.0)	2 (25.0)	2 (12.5)
Blood creatine phosphokinase increased	0 (0.0)	1 (16.7)	0 (0.0)	1 (8.3)	0 (0.0)	0 (0.0)	0 (0.0)	0 (0.0)
Blood triglycerides increased	0 (0.0)	0 (0.0)	0 (0.0)	0 (0.0)	0 (0.0)	0 (0.0)	1 (12.5)	1 (6.3)
Occult blood positive	0 (0.0)	0 (0.0)	0 (0.0)	0 (0.0)	1 (12.5)	0 (0.0)	1 (12.5)	1 (6.3)
**Infections and infestations**	0 (0.0)	0 (0.0)	1 (16.7)	1 (8.3)	0 (0.0)	1 (12.5)	0 (0.0)	1 (6.3)
Nasopharyngitis	0 (0.0)	0 (0.0)	1 (16.7)	1 (8.3)	0 (0.0)	0 (0.0)	0 (0.0)	0 (0.0)
Rhinitis	0 (0.0)	0 (0.0)	0 (0.0)	0 (0.0)	0 (0.0)	1 (12.5)	0 (0.0)	1 (6.3
**Injury, poisoning and procedural complications**	0 (0.0)	0 (0.0)	1 (16.7)	1 (8.3)	0 (0.0)	0 (0.0)	0 (0.0)	0 (0.0)
Ligament sprain	0 (0.0)	0 (0.0)	1 (16.7)	1 (8.3)	0 (0.0)	0 (0.0)	0 (0.0)	0 (0.0)
**Renal and urinary disorders**	0 (0.0)	0 (0.0)	1 (16.7)	1 (8.3)	0 (0.0)	0 (0.0)	0 (0.0)	0 (0.0)
Micturition urgency	0 (0.0)	0 (0.0)	1 (16.7)	1 (8.3)	0 (0.0)	0 (0.0)	0 (0.0)	0 (0.0)
**Ear and labyrinth disorders**	0 (0.0)	0 (0.0)	0 (0.0)	0 (0.0)	0 (0.0)	0 (0.0)	1 (12.5)	1 (6.3)
Ear pain	0 (0.0)	0 (0.0)	0 (0.0)	0 (0.0)	0 (0.0)	0 (0.0)	1 (12.5)	1 (6.3)

AE, adverse event; BID, twice daily; MRD, multiple-rising-dose; SRD, single-rising-dose.

Supplementary Table 4. Phase I study in healthy males: salient laboratory parameters

Salient laboratory parameters	SRD			MRD		
	**Placebo**	**BI 1015550 36 mg**	**BI 1015550 48 mg**	**Placebo**	**BI 1015550 6 mg BID**	**BI 1015550 12 mg BID**
**Number of subjects, n (%)**	6 (100.0)	6 (100.0)	6 (100.0)	8 (100.0)	8 (100.0)	8 (100.0)
**Haemoglobin in g/L, mean (SD)**						
Baseline	145.3 (24.8)	139.8 (11.9)	134.6 (12.1)	135.4 (22.1)	129.1 (10.0)	135.2 (9.0)
Last value on treatment	135.4 (30.0)	129.4 (10.3)	134.4 (10.4)	132.8 (21.9)	125.0 (6.2)	129.7 (9.9)
**Erythrocyte sedimentation rate in mm/h, mean (SD)**						
Baseline	7.9 (3.7)	5.8 (2.0)	6.7 (4.1)	8.1 (6.4)	12.2 (8.9)	6.9 (3.7)
Last value on treatment	8.3 (3.8)	10.0 (7.7)	8.3 (8.2)	10.6 (10.8)	13.8 (9.8)	10.3 (14.0)
**Fibrinogen in g/L, mean (SD)**						
Baseline	2.4 (0.3)	2.6 (0.8)	1.9 (0.8)	2.0 (0.5)	2.2 (0.6)	1.8 (0.5)
Last value on treatment	2.4 (0.7)	2.4 (0.9)	1.9 (0.8)	2.0 (0.6)	2.4 (0.5)	2.1 (0.9)

**Aspartate aminotransaminase in U/L, mean (SD)**						
Baseline	21.7 (5.1)	19.0 (3.5)	20.9 (4.5)	22.2 (5.1)	21.9 (3.1)	23.4 (7.0)
Last value on treatment	21.3 (2.3)	23.6 (14.4)	21.6 (4.0)	20.3 (3.0)	19.6 (3.5)	22.6 (6.2)
**Alanine aminotransaminase in U/L, mean (SD)**						
Baseline	24.3 (17.0)	15.6 (3.6)	18.1 (5.3)	15.5 (4.7)	14.9 (7.1)	27.7 (20.0)
Last value on treatment	21.1 (9.6)	16.1 (2.5)	19.3 (5.3)	18.9 (8.2)	14.7 (7.1)	21.9 (11.9)
**Creatinine kinase in U/L, mean (SD)**						
Baseline	419.7 (156.4)	342.7 (184.6)	263.4 (126.9)	393.6 (373.7)	368.8 (260.9)	273.4 (80.1)
Last value on treatment	368.2 (124.4)	1889.3[#] (3865.5)	229.4 (121.2)	279.4 (189.0)	309.0 (148.1)	745.6^ (1137.6)
**Total bilirubin in µmol/L, mean (SD)**						
Baseline	8.8 (6.5)	11.8 (6.2)	8.6 (2.7)	14.1 (11.3)	11.6 (5.5)	7.0 (4.1)
Last value on treatment	7.8 (3.6)	8.1 (3.3)	7.5 (3.1)	11.0 (5.0)	7.9 (3.8)	8.1 (2.9)
**High-sensitivity C-reactive protein in mg/L, mean (SD)**						
Baseline	3.8 (3.2)	1.3 (0.5)	2.5 (3.3)	3.4 (4.5)	4.6 (4.3)	1.4 (0.8)
Last value on treatment	3.3 (3.0)	1.6 (1.0)	1.9 (1.6)	3.5 (4.0)	9.8 (11.3)	2.6 (1.5)
**Triglycerides in mmol/L, mean (SD)**						

Baseline	1.1 (0.9)	1.7 (1.1)	1.7 (0.8)	1.2 (0.9)	0.9 (0.5)	2.3 (1.4)
Last value on treatment	1.1 (0.8)	1.7 (1.1)	1.8 (0.4)	1.5 (0.9)	0.9 (0.4)	2.3 (3.2)
**Urine pH, mean (SD)**						
Baseline	5.0 (0.0)	5.5 (0.8)	5.3 (0.5)	5.1 (0.4)	5.1 (0.4)	5.0 (0.0)
Last value on treatment	5.0 (0.0)	5.4 (0.6)	5.3 (0.5)	5.5 (0.5)	5.4 (0.5)	5.5 (0.5)

[#]Increased blood creatine phosphokinase was reported as an adverse event for one participant in the 36mg SRD group. This was attributed to physical exertion and was not considered drug related by the investigators. ^After the treatment period, increased creatine kinase was reported for one participant in the 12mg BID MRD group. This was attributed to physical stress at work and was not reported as an adverse event.

BID, twice daily; MRD, multiple-rising-dose; SD, standard deviation; SRD, single-rising-dose.

Supplementary Table 5. Phase Ic study in patients with IPF: baseline demographics

Demographic	Placebo	BI 1015550 18 mg BID	Total
**Number of patients (%)**	5 (100.0)	10 (100.0)	15 (100.0)
**Male, n (%)**	4 (80.0)	9 (90.0)	13 (86.7)
**Age in years, mean (SD)**	70.2 (3.3)	69.5 (10.1)	69.7 (8.3)
**Race, white n (%)**	5 (100.0)	10 (100.0)	15 (100.0)
**Weight in kg, mean (SD)**	81.1 (17.7)	83.1 (7.0)	82.5 (11.1)
**Smoking history n (%)**			
Never smoked	4 (80.0)	5 (50.0)	9 (60.0)
Former smoker	1 (20.0)	5 (50.0)	6 (40.0)
**Alcohol history n (%)**			
Never	1 (20.0)	1 (10.0)	2 (13.3)
Former	0 (0.0)	1 (10.0)	1 (6.7)
Current	4 (80.0)	8 (80.0)	12 (80.0)
**Time since first IPF diagnosis, median**	886.0	90.5	412.0
**FVC, mean (SD)**			
mL	3322.2 (1024.6)	3699.9 (1179.1)	3574.0 (1108.0)
% predicted	91.1 (17.1)	92.0 (15.3)	91.7 (15.3)
**FEV_1, mean (SD)**			
mL	2639.6 (763.8)	2957.9 (747.2)	2851.8 (741.4)
% predicted	94.5 (12.7)	97.4 (10.7)	96.4 (11.1)
**DL_{CO} Hb corrected**	62.4 (18.8)	61.6 (30.2)	61.9 (26.2)

BID, twice daily; DL_{CO}, diffusing capacity of the lung for carbon monoxide; FVC, forced vital capacity; FEV_1, forced expiratory volume in 1 second; Hb, haemoglobin; IPF, idiopathic pulmonary fibrosis; SD, standard deviation.

Supplementary Table 6. Phase Ic in patients with IPF: all AEs

System organ class/preferred term	Placebo n (%)	BI 1015550 18 mg BID n (%)	Total on treatment n (%)
**Number of subjects**	5 (100.0)	10 (100.0)	15 (100.0)
**Total with AEs**	5 (100.0)	10 (100.0)	15 (100.0)
**Nervous system disorders**	1 (20.0)	2 (20.0)	3 (20.0)
Headache	1 (20.0)	1 (10.0)	2 (13.3)
Somnolence	0 (0.0)	1 (10.0)	1 (6.7)
**Gastrointestinal disorders**	2 (40.0)	8 (80.0)	10 (66.7)
Diarrhoea	2 (40.0)	4 (40.0)	6 (40.0)
Flatulence	1 (20.0)	3 (30.0)	4 (26.7)
Anal fistula	0 (0.0)	1 (10.0)	1 (6.7)
Anal incontinence	0 (0.0)	1 (10.0)	1 (6.7)
Constipation	0 (0.0)	1 (10.0)	1 (6.7)
Faeces soft	0 (0.0)	1 (10.0)	1 (6.7)
Frequent bowel movements	0 (0.0)	1 (10.0)	1 (6.7)
Gastrointestinal sounds abnormal	0 (0.0)	1 (10.0)	1 (6.7)
Nausea	0 (0.0)	1 (10.0)	1 (6.7)
Proctalgia	0 (0.0)	1 (10.0)	1 (6.7)
**Investigations**	1 (20.0)	3 (30.0)	4 (26.7)
Occult blood positive	1 (20.0)	3 (30.0)	4 (26.7)
General physical condition worsened	0 (0.0)	1 (10.0)	1 (6.7)
**Infections and infestations**	2 (40.0)	5 (50.0)	7 (46.7)
Nasopharyngitis	0 (0.0)	4 (40.0)	4 (26.7)
Pharyngitis	1 (20.0)	0 (0.0)	1 (6.7)
Respiratory tract infection	1 (20.0)	0 (0.0)	1 (6.7)
Bronchitis	0 (0.0)	1 (10.0)	1 (6.7)

Oral herpes	0 (0.0)	1 (10.0)	1 (6.7)
**Injury, poisoning and procedural complications**	0 (0.0)	1 (10.0)	1 (6.7)
Scar	0 (0.0)	1 (10.0)	1 (6.7)
**Musculoskeletal and connective tissue disorders**	0 (0.0)	6 (60.0)	6 (40.0)
Muscle spasms	0 (0.0)	2 (20.0)	2 (13.3)
Muscle tightness	0 (0.0)	2 (20.0)	2 (13.3)
Pain in extremity	0 (0.0)	2 (20.0)	2 (13.3)
Spinal pain	0 (0.0)	1 (10.0)	1 (6.7)
Temporomandibular joint syndrome	0 (0.0)	1 (10.0)	1 (6.7)
Trigger finger	0 (0.0)	1 (10.0)	1 (6.7)
**General disorders and administration site conditions**	1 (20.0)	3 (30.0)	4 (26.7)
Fatigue	1 (20.0)	2 (20.0)	3 (20.0)
General physical health deterioration	0 (0.0)	1 (10.0)	1 (6.7)
**Eye disorders**	1 (20.0)	1 (10.0)	2 (13.3)
Vision blurred	1 (20.0)	0 (0.0)	1 (6.7)
Conjunctival hyperaemia	0 (0.0)	1 (10.0)	1 (6.7)
**Psychiatric disorders**	0 (0.0)	2 (20.0)	2 (13.3)
Insomnia	0 (0.0)	2 (20.0)	2 (13.3)
**Respiratory, thoracic and mediastinal disorders**	1 (20.0)	1 (10.0)	2 (13.3)
Dyspnoea exertional	1 (20.0)	0 (0.0)	1 (6.7)
Dyspnoea	0 (0.0)	1 (10.0)	1 (6.7)
**Skin and subcutaneous tissue disorders**	1 (20.0)	2 (20.0)	3 (20.0)

Skin odour abnormal	1 (20.0)	0 (0.0)	1 (6.7)
Pruritus	0 (0.0)	1 (10.0)	1 (6.7)
Rash	0 (0.0)	1 (10.0)	1 (6.7)
**Cardiac disorders**	0 (0.0)	1 (10.0)	1 (6.7)
Bradycardia	0 (0.0)	1 (10.0)	1 (6.7)

AE, adverse event; BID, twice daily; IPF, idiopathic pulmonary fibrosis.

Supplementary Table 7. Phase Ic in patients with IP: salient laboratory parameters

	Placebo	BI 1015550 18 mg BID
**Number of subjects, n (%)**	5 (100.0)	10 (100.0)
**Haemoglobin in g/L, mean (SD)**		
Baseline	153.7 (13.9)	140.6 (10.7)
Last value on treatment	143.1 (16.9)	132.4 (13.8)
**Erythrocyte sedimentation rate in mm/h, mean (SD)**		
Baseline	14.7[#] (12.1)	20.9 (12.9)
Last value on treatment	16.3 (9.8)	29.1 (21.5)
**Fibrinogen in g/L, mean (SD)**		
Baseline	3.6 (0.5)	4.0 (0.8)
Last value on treatment	3.6 (0.5)	4.2 (0.8)
**Aspartate aminotransferase in U/L, mean (SD)**		
Baseline	21.3 (13.0)	18.3 (7.2)
Last value on treatment	20.4 (11.8)	14.4 (6.3)
**Alanine aminotransferase in U/L, mean (SD)**		
Baseline	24.3 (14.7)	14.6 (5.2)
Last value on treatment	22.9 (16.6)	12.7 (3.0)
**Creatinine in U/L, mean (SD)**		
Baseline	79.9 (8.3)	86.8 (14.4)
Last value on treatment	81.5 (14.1)	86.4 (25.1)

**Bilirubin in µmol/L, mean (SD)**		
Baseline	7.5 (1.4)	9.5 (2.6)
Last value on treatment	7.7 (2.8)	8.0 (2.1)
**High-sensitivity C-reactive protein in mg/L, mean (SD)**		
Baseline	1.2 (0.8)	2.8 (2.5)
Last value on treatment	2.0 (1.3)	6.0 (9.9)
**Triglycerides in mmol/L, mean (SD)**		
Baseline	1.7 (1.1)	0.7 (0.3)
Last value on treatment	0.9 (0.24)	0.5 (0.1)
**Urine pH, mean (SD)**		
Baseline	5.7 (0.8)	5.5 (0.2)
Last value on treatment	5.7 (0.6)	5.6 (0.6)

[#]values available for n=4 patients in the placebo group. BID, twice daily; IPF, idiopathic pulmonary fibrosis; SD, standard deviation.
